# Molecularly Targeted Cancer Medications and Kidney Health

**DOI:** 10.1001/jamanetworkopen.2025.41221

**Published:** 2025-11-06

**Authors:** Susan Ziolkowski, Jin Long, Yutong Zhong, Hannah Morgan-Cooper, Heather Wakelee, Ali Khaki, Fauzia Riaz, Sunil Reddy, Mary Leonard, Shuchi Anand

**Affiliations:** 1 Department of Medicine, Nephrology, Stanford University, Stanford, California; 2 Department of Pediatrics, Stanford University, Stanford, California; 3 Stanford Healthcare, Stanford University, Stanford, California; 4 Department of Medicine, Oncology, Stanford University, Stanford, California

## Abstract

**Importance:**

Oral molecularly targeted cancer medications are widely used. Product labels describe an increase in serum creatinine (sCr), potentially due to reversible inhibition of tubular creatinine secretion rather than acute kidney injury.

**Objective:**

To determine the 2-year incidence and relative rates of progressive kidney dysfunction in patients treated with molecularly targeted cancer medications associated with an acute change in sCr handling (pseudoacute kidney injury).

**Design, Setting, and Participants:**

Retrospective cohort study of adult patients throughout the San Francisco Bay area treated at Stanford Hospital and clinics since 2008 receiving cyclin dependent kinase 4 or 6 (CDK4/6) inhibitors, poly(adenosine diphosphate-ribose) polymerase inhibitors, and specific drugs within tyrosine kinases inhibitor subgroups with an available estimated glomerular filtration rate (eGFR) from days 1 to 60 after drug start compared with propensity score–matched patients without cancer.

**Exposure:**

Molecularly targeted cancer medications associated with an acute change in sCr.

**Main outcomes and measures:**

Progressive kidney dysfunction defined as a sustained 30% or more decline in eGFR or reaching end-stage kidney disease, accounting for a potentially reversible rise in sCr at drug start by defining baseline eGFR as the mean value from days 1 to 60 after drug start.

**Results:**

Among 5015 patients in the treated cohort, the median (IQR) age was 62 (51-72) years, 3264 (65%) were female, and the mean (SD) baseline eGFR before drug start was 87 (23) mL/min/1.73m^2^. Using cox proportional hazards regression models, the overall incidence and rate of progressive kidney dysfunction was higher in the treated cohort than in propensity-matched cohorts of patients without cancer (44 vs 38 per 1000 person-years; hazard ratio [HR], 1.4; 95% CI, 1.2-1.6). Rates of kidney dysfunction were higher among patients treated with CDK4/6 inhibitors (HR, 1.9; 95% CI, 1.4-2.6), epidermal growth factor receptor inhibitors (HR, 1.8; 95% CI, 1.2-2.5), vascular endothelial growth factor receptor inhibitors (HR, 2.1; 95% CI, 1.6-2.7), and B-Raf inhibitors (HR, 1.9; 95% CI, 1.1-3.3) compared with controls. These higher rates persisted in patients without a 20% or more increase in sCr at drug start and without exposure to chemotherapy or immunotherapy.

**Conclusions and relevance:**

In this cohort study of patients treated with molecularly targeted anticancer medications associated with an acute rise in sCr, we observed higher incidence and relative rate for kidney dysfunction among treated patients, compared with matched cancer-free patients.

## Key Points

**Question:**

Do patients treated with oral molecularly targeted cancer medications that are associated with an acute change in serum creatinine handling (pseudoacute kidney injury) have higher rates of progressive kidney dysfunction over 2 years?

**Findings:**

This cohort study found a higher incidence and relative rate for progressive kidney dysfunction for 5015 patients treated with cyclin dependent kinase 4 or 6 (CDK4/6) inhibitors, vascular endothelial growth factor receptor (VEGFR), and epidermal growth factor receptor (EGFR) inhibitors compared with matched controls without cancer. B-Raf (BRAF) inhibitors also had a higher relative rate. Breakpoint cluster region-Abelson leukemia virus 1, poly(adenosine diphosphate-ribose) polymerase inhibitors, human epidermal growth factor receptor 2, and anaplastic lymphoma kinase inhibitors did not have increased rates.

**Meaning:**

These findings suggest that clinicians may need to monitor for progressive kidney dysfunction for patients treated with CDK4/6 inhibitors, EGFR, VEGFR, and BRAF inhibitors.

## Teaser

This cohort study evaluates the 2-year incidence and relative rates of progressive kidney dysfunction in patients treated with molecularly targeted cancer medications.

## Introduction

Oral molecularly targeted medications are first-line treatments for a range of cancer and hematologic conditions with potential for prolonged efficacy.^1^ Limited data exist on the effects of these medications on kidney function over time, although many—including inhibitors of cyclin dependent kinase 4 or 6 (CDK4/6) inhibitors, poly(adenosine diphosphate-ribose) polymerase (PARP) inhibitor, and specific tyrosine kinases (TKIs)—are known to cause an early rise in serum creatinine (sCr).^1,2,3,4,5^ For example, product labels of crizotinib, an anaplastic lymphoma kinase (ALK) inhibitors, report an increase in sCr in 99% of patients.^6^ Subsequent studies report a median 21.2% increase in sCr from baseline within 2 weeks of initiating therapy.^7^

One explanation for the early rise in sCr observed at drug initiation is inhibition of tubular creatinine secretion through organic anion transporters (OAT), organic cation transporters (OCT), and multidrug and toxin extrusion protein (MATE) transporters.^8,9,10,11^ Therefore, higher sCr may not reflect reduced glomerular filtration rate (GFR) but rather decreased secretion of creatinine, similar to the phenomenon observed with cimetidine. Within 6 hours of cimetidine start, studies demonstrate an increase in sCr.^12,13^ However, these same medications inhibit cell growth and repair, and are associated with biopsy-proven kidney disease. The largest case series of 6 kidney biopsies from patients with acute kidney injury while treated with CDK4/6 inhibitors demonstrated predominantly acute tubular necrosis, accompanied by electrolyte disturbances.^14^ Additional case reports for other drug classes suggest glomerular and interstitial kidney injuries.^15,16,17,18,19^ Overall, data are limited to case reports and single-center small studies^3,5,20,21,22,23^ with limited follow-up. In patients with cancer, development or progression of kidney dysfunction may limit future therapeutic options, and a 10-mL/min/1.73 m^2^ lower estimated GFR (eGFR) is associated with 18% higher mortality.^24^ Therefore, clinicians require a better understanding of the effects of molecularly targeted medications on kidney health so they can be used more safely and effectively.

Using Stanford electronic health record (EHR) data from patients treated with the CDK4/6 inhibitors, PARP inhibitor, and select drugs from 7 classes of TKIs that are all associated with an acute change in sCr handling, we determined the incidence rates of progressive kidney dysfunction (defined as a sustained ≥30% decline in eGFR^25^ or reaching end-stage kidney disease [ESKD]). We compared these incidence rates with propensity score–matched cohorts without cancer. We hypothesized that patients treated with CDK4/6 inhibitors, PARP inhibitors, and specific TKIs would have higher rates and an increased hazard of progressive kidney dysfunction compared with the matched cohorts independent of other nephrotoxic exposures, and that a larger change in sCr at drug initiation would be associated with higher risk of progressive kidney dysfunction.

## Methods

We conducted a retrospective longitudinal cohort study in the Stanford Medicine Research Data Repository Observational Medical Outcomes Partnership (OMOP) including EHR data from approximately 4 million patients seen within the Stanford Hospital, Tri-Valley Hospital, and outpatient clinics throughout the San Francisco Bay Area since 2008. The study received institutional review board approval from Stanford University. The requirement for informed consent was waived because because data were deidentified, in accordance with 45 CFR §46. We followed the Strengthening the Reporting of Observational Studies in Epidemiology (STROBE) reporting guidelines for cohort studies.^26^

The OMOP Common Data Model systematically transforms data from observational databases into a common format (data model) with common terminologies, vocabularies, and coding schemes.^27^ OMOP concept identifiers are standardized medical codes for diagnoses, procedures, medication orders, and laboratory test orders (*International Statistical Classification of Diseases and Related Health Problems, Tenth Revision (ICD-10)*, *Current Procedural Terminology* or Healthcare Common Procedure Coding System, RxNorm Concept Unique Identifier, and Logical Observation Identifiers Names and Codes, respectively).

### Study Design and Population

**Figure 1.**
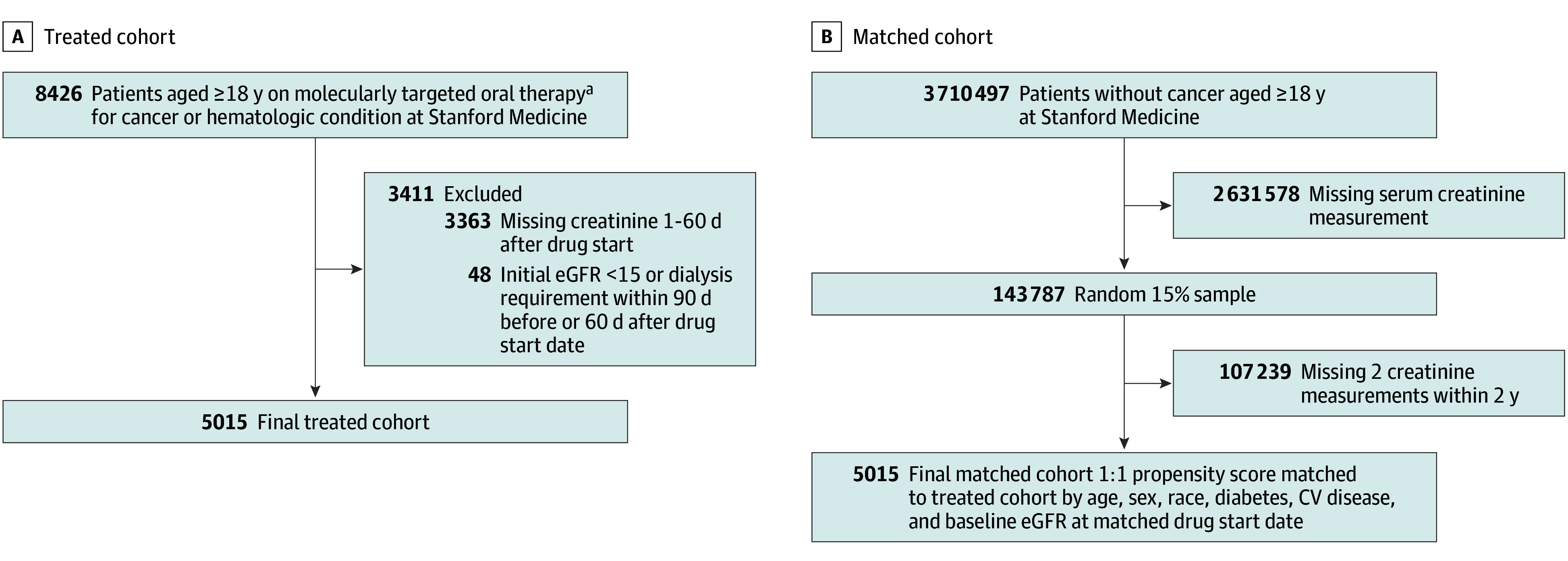
Cohort Generation of Treated Cohort and Matched Cohort A, Cohort generation of the treated cohort starting with extraction of patients ages 18 years and older at Stanford Medicine treated with cancer medications that are associated with an increase in serum creatinine at drug start or decreased creatinine secretion (8426 patients). We removed patients without a serum creatinine measurement between day 1 and 60 after drug start (3363 patients), and patients who already had an eGFR of 15 or less or required dialysis within 90 days before or within the first 60 days of drug start date (48 patients). This resulted in a final treated cohort of 5015 patients. B, Cohort generation of the matched cohort. Our starting cohort consisted of all patients at Stanford Medicine ages 18 years or older without any cancer concept identification (3 710 497 patients). We removed patients without any serum creatinine measurement (2 631 578 patients). Of those patients, we created a random 15% sample to allow more manageable computation (161 838 patients). We then removed patients from that sample without at least 2 serum creatinine measurements (107 239 patients) within any 2-year time frame. We then propensity score–matched the remaining 54 598 patients to the treated cohort by age, sex, race, diabetes, cardiovascular disease, and baseline eGFR at time of matched drug start date.

Our primary analytic cohort is composed of patients treated with CDK4/6 inhibitors, PARP inhibitors, and select drugs from 7 classes of TKIs, all of which are associated with an acute change in sCr handling (eTable 1 in Supplement 1). We included patients 18 years or older with at least 1 sCr value available between 1 to 60 days after drug start defined as the date of first drug prescription (Figure 1A). We excluded patients with ESKD, or eGFR values less than 15 mL/min/1.73 m^2^ within 90 days before or 60 days after drug start. This defined our treated cohort. Among the treated cohort, 176 patients were exposed to 2 or more drugs classes within the 2-year incidence period and were classified according to their first drug exposure. Our comparator cohort included propensity score–matched patients without previous or current cancer (Figure 1B).

### Exposure and Covariates

We created concept sets for laboratory measurements, demographics, medications, comorbidities, and cancer types.^28^ We used creatinine-based 2021 Chronic Kidney Disease Epidemiology Collaboration (CKD-EPI) equation to calculate eGFR.^29^ Race was categorized according to designations in the EHR: White, Black, Asian, other, and unknown. Other race is what is recorded in the EHR as a race not in the specified categories but is not unknown. Race was assessed in this study, consistent with other studies, because it may confound the association between medication exposure and kidney outcomes.

We defined baseline eGFR as the mean eGFR within 1 to 60 days after the drug start date (eFigure 1 in Supplement 1). We also report sCr and eGFR values before drug start. We calculated the change in sCr by subtracting values before drug start from the mean of values within 1 to 60 days after drug start. Based on the expected 15% to 30% change in sCr with inhibition of tubular creatinine secretion and intraindividual variability of the sCr assay, we selected a 20% or more early change in sCr as an exposure.^30,31,32^ Other exposures of interest included genitourinary cancer and concurrent potentially nephrotoxic exposures—chiefly, proton pump inhibitors (PPIs), immunotherapy, and specific chemotherapy (cisplatin, pemetrexed, gemcitabine, and bevacuzimab)—and for these, we extracted dates of drug administration.

For descriptive purposes, we determined cancer type and baseline urine measurements (eMethods 1-2 in Supplement 1). Urine measurements and cystatin C levels were available in 45% and 1.2% of patients, respectively; we did not include these measures in further modeling.

#### Outcome

We defined progressive kidney dysfunction based on previous meta-analysis by Coresh et al^33^ of a collection of cohorts with cardiac and kidney outcomes that demonstrated a 30% or more decline in eGFR over 2 years was associated with 5- to 7-fold higher relative rate of ESKD and 2-fold higher relative rate for mortality compared with no change in eGFR.^33^ Using the same definition,^33^ our primary outcome was one of the following occurring within 2 years after the baseline eGFR: (1) 30% or more drop in eGFR or eGFR<10 mL/min/1.73m^2^ confirmed on laboratory tests 90 or more days later; (2) 30% or more drop in eGFR or eGFR<10 mL/min/1.73m^2^ without additional data before censor date; or (3) need for dialysis. Those in the primary cohort without eGFR values after 60 days of drug start were defined with missing progressive kidney dysfunction status. We used multiple imputation (see Statistical Analysis section) to impute their values. We ascribed the event date to the first occurrence of our primary outcome and obtained death dates via EHR.^34^

### Statistical Analysis

We categorized patients in the treated cohort into 9 class categories. We employed a logistic regression model to calculate the propensity score for each patient in the treated cohort for the following covariates: age, sex, diabetes, ischemic heart disease, congestive heart failure, and eGFR value nearest to, but preceding, the drug start date. We used a nearest neighbor 1:1 matching algorithm to pair each patient in the treated cohort with a cancer-free patient with the most similar propensity score. To assess covariate balance, we calculated standardized mean differences for all covariates after matching.

We determined incidence rates for progressive kidney dysfunction within the matched and treated cohort, overall and grouped by class. We employed cox proportional hazard models under the Fine and Gray specification^35^ to evaluate incidence in treated vs matched cohort, overall and for each drug subgroup, while accounting for competing risk of death and established risk factors for CKD (age, sex, race or ethnicity, diabetes, ischemic heart disease, congestive heart failure, chronic obstructive pulmonary disease [COPD], cirrhosis, body mass index, and eGFR before drug start). We censored patients treated with the date of their last health care encounter or end of study period.

We used multivariate cox proportional hazard models to determine if potential risk factors such as genitourinary cancer, exposure to nephrotoxic chemotherapy or immunotherapy, PPIs, and early changes in sCr were associated with our outcome. We categorized cancer drugs based on mechanism of kidney injury (ie, gemcitabine with bevacizumab and cisplatin with pemetrexed).

To determine the relative rate of progressive kidney dysfunction associated with treatment compared with the matched cohort, we computed cause-specific hazard models^36^ and 95% CIs adjusting for established risk factors. In separate models, we present estimates for the overall cohort and each class. We also performed subgroup analyses by risk factors for progressive kidney dysfunction.

We used multiple imputation for the following missing data in our analytic cohort: follow up sCr and time to outcome in patients without any values following day 60 (267 patients [5.3%]), race (21 patients [0.4%]), and baseline BMI (133 patients [2.7%]). We assumed missingness at random and used R package mice to generate imputed datasets. We then used the pool() function from the mice package to combine the results from the analyses performed on each imputed dataset. All analyses were conducted using R software version 4.4.1 (R Project for Statistical Computing).

## Results

### Patient Characteristics

Among 5015 patients in the treated cohort, the median (IQR) age was 62 (51-72) years, 3264 (65%) were female, and the mean (SD) baseline eGFR before drug start was 87 (23) mL/min/1.73 m^2^. The most common drug class was CDK4/6 inhibitors (1194 patients) and vascular endothelial growth factor receptor (VEGFR) inhibitors (1131 patients) (Table 1). Diabetes prevalence varied from 11.5% among patients treated with ALK inhibitors (26 patients) to 26.7% among patients treated with MET inhibitors (8 patients). Patients had a median (IQR) of 3 (1-5) sCr values in days 1 to 60 of drug start. There were 443 patients (8.8%) for whom baseline sCr was based only on values within day 1 to 14 days of drug start.

**Table 1.** Baseline Characteristics of Patients Treated With Oral Molecularly Targeted Cancer Medications (Treated Cohort)

Characteristic	Drug class (inhibitors)								
	CDK4/6 (n = 1194)	PARP (n = 570)	ALK (n = 226)	*BCR-ABL* (n = 590)	EGFR (n = 774)	VEGFR (n = 1131)	*ERBB2* (n = 218)	BRAF (n = 282)	MET (n = 30)
Age, median (IQR), y	61 (40-82)	63 (44-82)	60 (40-80)	58 (34-82)	69 (52-86)	61 (42-80)	55 (39-71)	62 (41-83)	73 (58-88)
Male	73 (6.1)	82 (14.4)	86 (38.1)	322 (54.6)	264 (34.1)	716 (63.3)	24 (11.0)	166 (58.9)	18 (60.0)
Female	1121 (93.9)	488 (85.6)	140 (61.9)	268 (45.4)	510 (65.9)	415 (36.7)	194 (89.0)	116 (41.1)	12 (40.0)
Race^a^									
Asian	286 (24.0)	144 (25.3)	104(46.0)	135 (22.9)	438 (56.6)	286 (25.3)	66 (30.3)	47 (16.7)	7 (23.3)
Black	29 (2.4)	16 (2.8)	6 (2.7)	18 (3.1)	18 (2.3)	38 (3.4)	11 (5.0)	2 (0.7)	0
White	670 (56.1)	324 (56.8)	83 (36.7)	312 (52.9)	222 (28.7)	569 (50.3)	100(45.9)	183(64.9)	16 (53.3)
Other^b^	204 (17.1)	85 (14.9)	31 (13.7)	123 (20.8)	96 (12.4)	232 (20.5)	39 (17.9)	47 (16.7)	7 (23.3)
Unknown	5 (0.4)	1 (0.2)	2 (0.9)	2 (0.3)	0	6 (0.5)	2 (0.9)	3 (1.1)	0
Comorbidities									
Hypertension	432 (36.2)	261 (45.8)	86 (38.1)	213 (36.1)	352 (45.5)	537 (47.5)	74 (33.9)	135 (47.9)	16 (53.3)
Diabetes	167 (14.0)	92 (16.1)	26 (11.5)	98 (16.6)	126 (16.3)	261 (23.1)	32 (14.7)	40 (14.2)	8 (26.7)
Ischemic heart disease	79 (6.6)	47 (8.2)	20 (8.8)	46 (7.8)	76 (9.8)	73 (6.5)	9 (4.1)	27 (9.6)	8 (26.7)
Congestive heart failure	45 (3.8)	21 (3.7)	11 (4.9)	44 (7.5)	32 (4.1)	58 (5.1)	15 (6.9)	17 (6.0)	1 (3.3)
Chronic obstructive pulmonary disease	32 (2.7)	28 (4.9)	14 (6.2)	25 (4.2)	49 (6.3)	64 (5.7)	4 (1.8)	16 (5.7)	1 (3.3)
Cirrhosis	15 (1.3)	10 (1.8)	1 (0.4)	12 (2.0)	10 (1.3)	170 (15.0)	2 (0.9)	3 (1.1)	0
Genitourinary cancer	54 (4.5)	103 (18.1)	27 (11.9)	26 (4.4)	71 (9.2)	371 (32.8)	13 (6.0)	39 (13.8)	11 (36.7)
Measurements, mean (SD)									
Body mass index^a^^,^^c^	27.4 (6.6)	27.0 (19.5)	25.0 (4.9)	27.1 (6.1)	24.6 (8.0)	27.1 (8.0)	25.6 (5.6)	26.6 (5.8)	24.7 (4.3)
Serum albumin, g/dL^a^	3.94 (0.55)	3.83(0.57)	3.54 (0.71)	3.66 (0.67)	3.77 (0.59)	3.41 (0.71)	3.63 (0.56)	3.51 (0.75)	3.44 (0.59)
Serum creatinine, mg/dL^d^	0.8 (0.3)	0.8(0.3)	0.9 (0.5)	1.0 (0.4)	0.8 (0.4)	1.0 (0.4)	0.8 (0.2)	0.9 (0.4)	0.9 (0.4)
eGFR, mL/min/1.73 m^2^	89.8 (22.0)	90.3 (21.4)	88.0 (22.7)	84.0 (24.9)	86.2 (20.1)	83.3 (26.0)	93.2 (21.4)	90.3 (23.7)	83.7 (25.2)
eGFR categories, mL/min/1.73 m^2^									
≥90	695 (58.2)	342 (60.0)	124 (54.9)	259 (43.9)	401 (51.8)	510 (45.1)	130 (59.6)	161 (57.1)	15 (50.0)
60-89	362 (30.3)	172 (30.2)	75 (33.2)	237 (40.2)	283 (36.6)	372 (32.9)	67 (30.7)	88 (31.2)	9 (30.0)
45-59	82 (6.9)	35 (6.1)	17 (7.5)	50 (8.5)	57 (7.4)	162 (14.3)	16 (7.3)	21 (7.4)	4 (13.3)
<45	51 (4.3)	21 (3.7)	10 (4.4)	44 (7.5)	33 (4.3)	87 (7.7)	5 (2.3)	12 (4.3)	2 (6.7)

Abbreviations: ALK, anaplastic lymphoma kinase; BRAF, B-Raf proto-oncogene, serine/threonine kinase; CDK4/6, cyclin dependent kinase 4 or 6; eGFR, estimated glomerular filtration rate calculated using CKD-EPI 2021 creatinine equation; EGFR, epidermal growth factor receptor; *ERBB2*, human epidermal growth factor receptor 2; MET, mesenchymal-epithelial transition inhibitor; PARP, poly(adenosine diphosphate-ribose) polymerase; VEGFR, vascular endothelial growth factor receptor.

SI conversion factors: to convert serum albumin to g/L, multiply by 10; creatinine to µmol/L, multiply by 76.25.

^a^ Race available in 4994 patients, body mass index available in 4882 patients, serum albumin available in 4817 patients.

^b^ Other race is recorded in the electronic health record as a race not in the specified categories but is not unknown.

^c^ Calculated as weight in kilograms divided by height in meters squared.

^d^ All serum creatinine and eGFR values obtained within 1 year before drug start.

eTable 2 in Supplement 1 shows the type of cancers that were associated with each class. Baseline urine measurements are shown in eTable 3 in Supplement 1.

Baseline characteristics of the propensity score–matched cohort are in eTable 4 in Supplement 1. Standardized mean differences found minimal imbalances between the treated and matched cohort (all ≤0.1).

### Exposures

eTable 5 in Supplement 1 shows the drug duration for each class, change in sCr at drug start, and other medication exposures over 2 years following drug start date. A total of 2053 patients (40.9%) were treated with their assigned class for the entire 2-year follow-up. Median (IQR) drug exposure was 21 (11-24) months. Among patients not receiving their allotted drug for the full 2 years, a majority of this group was censored for reaching the outcome or removed for competing risk of death. A total of 672 patients (13.4%) contributed some time off drug within the analyses. Among those 672 patients, the median (IQR) time of follow up time off drug was 65 (60-131) days. Overall, 1059 patients (21.1%) had a 20% or greater change in sCr at drug start, most common among patients treated with CDK4/6 inhibitors. PPIs were the most common medication exposure. Fifty-six patients had a partial or full nephrectomy during the follow-up period. The treated group had more hospitalizations compared with the matched group (2156 [43%] vs 1204 [24%]).

### Incidence Rates of Progressive Kidney Dysfunction, Overall and by Class

**Figure 2.**
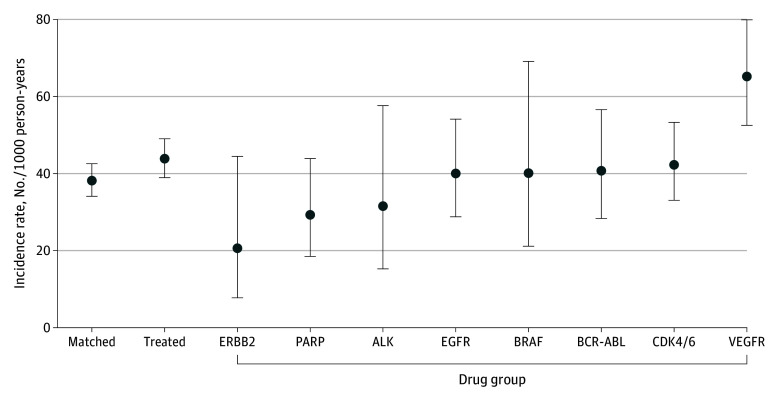
Incidence Rates of Progressive Kidney Dysfunction in Treated and Matched Cohorts The incidence rate for our primary outcome (accounting for death and lost to follow-up) per 1000 person-years is shown on the y-axis. Drug classes on the x-axis refer to inhibitors. Due to wide confidence intervals, MET inhibitors results are reported in the text and not displayed here. MET inhibitors are included in overall treated group. ALK indicates anaplastic lymphoma kinase; BRAF, B-Raf proto-oncogene, serine/threonine kinase; CDK4/6, cyclin dependent kinase 4 or 6; EGFR, epidermal growth factor receptor; *ERBB2*, human epidermal growth factor receptor 2; PARP, poly(adenosine diphosphate-ribose) polymerase; VEGFR, vascular endothelial growth factor receptor.

**Figure 3.**
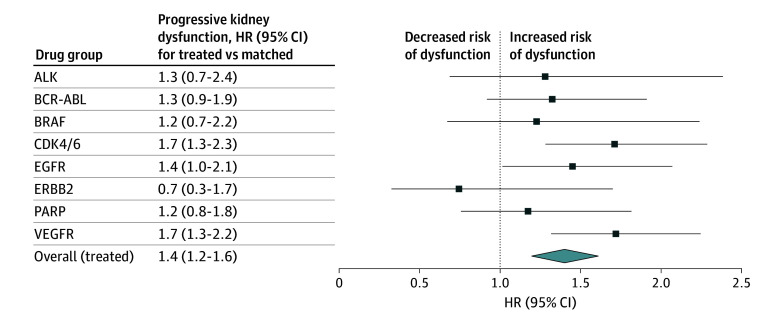
Adjusted Subdistribution Hazard Ratios for Progressive Kidney Dysfunction Within Each Drug Class as Compared With Propensity-Matched Cohort Without Cancer The subdistribution hazard ratios for progressive kidney dysfunction are shown for each drug group compared with their respective propensity score–matched cohort. The blue dots represent the hazard ratio and 95% CIs. Due to wide confidence intervals, MET inhibitors results reported in the text and not displayed here. MET inhibitors are included in overall treated group. Model is adjusted for hypertension, diabetes, ischemic heart disease, congestive heart failure, chronic obstructive pulmonary disease, cirrhosis, age, sex, race, body mass index, and eGFR before drug start. ALK indicates anaplastic lymphoma kinase; BRAF, B-Raf proto-oncogene, serine/threonine kinase; CDK4/6, cyclin dependent kinase 4 or 6; EGFR, epidermal growth factor receptor; *ERBB2*, human epidermal growth factor receptor 2; PARP, poly(adenosine diphosphate-ribose) polymerase; VEGFR, vascular endothelial growth factor receptor.

Over a median (IQR) of 561 (257-730) days of follow-up, incidence of progressive kidney dysfunction was higher among the treated group compared with the matched cohort (44 vs 38 per 1000 person-years; hazard ratio [HR], 1.4; 95% CI, 1.2-1.6) (Figure 2). The median (IQR) time to event was 194 (108-369) days. Incidence rates were highest in patients treated with VEGFR inhibitors and CDK4/6 inhibitors; incidence was lowest in patients treated with *ERBB2* inhibitors (also known as HER2 inhibitors) (eTable 6 in Supplement 1). Adjusted subdistribution HR confirmed these findings, and patients treated with epidermal growth factor receptor (EGFR) inhibitors also had higher incidence of progressive kidney dysfunction (Figure 3; eTable 7 in Supplement 1). The incidence rate for patients treated with MET inhibitors was 120 per 1000 person-years and subdistribution HR was 4.2 (95% CI, 1.6-11.1). Sensitivity analysis removing patients who received a partial or full nephrectomy revealed similar findings.

### Risk Factors for Progressive Kidney Dysfunction Within Treated Cohort, by Class

Exposure to gemcitabine or bevacizumab, PPIs, and immunotherapy were positively associated with progressive kidney dysfunction (eTable 8 in Supplement 1). Change in sCr at drug start as a categorical 20% or more change or as a continuous variable was not associated with progressive kidney dysfunction.

### Relative Rates for Progressive Kidney Dysfunction Compared With Propensity Score–Matched Cohort Without Cancer

Accounting for established risk factors for CKD, the relative rate of progressive kidney dysfunction was higher than in matched controls for patients treated with CDK4/6 inhibitors (HR, 1.9; 95% CI, 1.4-2.6), EGFR inhibitors (HR, 1.8; 95% CI, 1.2-2.5), VEGFR inhibitors (HR, 2.1; 95% CI, 1.6-2.7), B-Raf (BRAF) inhibitors (HR, 1.9; 95% CI, 1.1-3.3), and MET inhibitors (HR, 5.3; 95% CI, 1.9-14.5) (Table 2). In subgroup analyses, patients treated with these classes continued to experience higher relative rate of progressive kidney dysfunction in the absence of hospitalizations, exposure to PPIs, nephrotoxic chemotherapy or immunotherapy, or an early 20% or more increase in sCr at drug start. Due to the small cohort size of MET inhibitors, subgroup analyses were not performed in this group.

**Table 2.** Cause-Specific Hazard Ratios (HR) for Risk of Progressive Kidney Dysfunction in the Treated Cohort Compared With the Matched Cohort by Drug Class

Characteristic	Drug class (inhibitors), HR (95% CI)^a^								
	CDK4/6	PARP	ALK	*BCR-ABL*	EGFR	VEGFR	*ERBB2*	BRAF	Overall
Entire cohort	1.9 (1.4-2.6)	1.3 (0.8-2.1)	1.4 (0.71-2.60)	1.4 (1.0-2.0)	1.8 (1.2-2.5)	2.1 (1.6-2.7)	1.0 (0.5-2.2)	1.9 (1.1-3.3)	1.6 (1.4-2.0)
Subgroup analyses									
≥20% Change in sCr^c^									
Present	1.8 (1.1-3.0)	0.8 (0.3-2.5)	1.2 (0.3-5.0)	2.7 (1.4-5.2)	1.7 (0.8-3.7)	2.8 (1.6-4.9)	NA^c^	4.3 (1.6-11.8)	1.9 (1.4-2.6)
Absent	2.0 (1.5-2.8)	1.6 (0.9-2.4)	1.4 (0.7-2.9)	1.2 (0.8-1.9)	1.8 (1.2-2.6)	2.0 (1.5-2.6)	1.3 (0.6-2.7)	1.6 (0.8-3.0)	1.6 (1.4-2.0)
Proton pump inhibitor									
Yes	1.4 (0.9-2.3)	1.2 (0.6-2.5)	1.5 (0.6-3.7)	2.0 (1.3-3.1)	2.3 (1.4-3.8)	2.9 (2.1-3.9)	0.8 (0.2-3.1)	1.6 (0.7-3.6)	2.0 (1.6-2.4)
No	2.3 (1.6-3.1)	1.4 (0.8-2.4)	1.3 (0.5-3.1)	0.8 (0.4-1.6)	1.5 (0.9-2.4)	1.3 (0.8-2.0)	1.2 (0.5-3.0)	2.3 (1.1-4.7)	1.5 (1.2-1.9)
Immunotherapy									
Yes	3.3 (1.3-8.1)	1.9 (0.8-4.5)	NA^c^	NA^c^	1.3 (0.3-5.1)	1.7 (0.9-3.2)	NA^c^	2.3 (0.9-5.7)	1.9 (1.2-2.8)
No	1.9 (1.4-2.5)	1.2 (0.7-2.0)	1.4 (0.7-2.7)	1.4 (1.0-2.1)	1.8 (1.3-2.6)	2.1 (1.6-2.8)	1.1 (0.5-2.3)	1.7 (0.9-3.4)	1.6 (1.4-2.0)
Cisplatin and/or pemetrexed									
Yes	24.3 (3.3-179.2)	NA^c^	NA^c^	NA^c^	2.4 (1.4-4.3)	NA^c^	2.4 (1.4-4.3)	NA^c^	2.1 (1.2-3.7)
No	2.0 (1.4-2.5)	1.3 (0.8-2.1)	1.5 (0.8-2.9)	1.4 (1.0-2.0)	1.6 (1.0-2.4)	2.1 (1.6-2.7)	1.0 (0.5-2.2)	2.0 (1.1-3.4)	1.6 (1.4-2.0)
Gemcitabine and/or bevacizumab									
Yes	1.4 (0.5-4.4)	1.6 (0.7-3.6)	1.6 (0.2-11.5)	NA^c^	1.4 (0.5-3.9)	2.9 (1.4-5.9)	NA^c^	3.2 (0.5-23.1)	1.8 (1.1-2.7)
No	2.0 (1.5-2.7)	1.3 (0.7-2.1)	1.3 (0.7-2.6)	1.4 (1.0-2.1)	1.8 (1.3-2.6)	2.0 (1.5-2.7)	1.1 (0.5-2.4)	1.8 (1.0-3.3)	1.7 (1.4-2.0)
Immunotherapy or above chemotherapy									
Yes	2.0 (1.5-2.7)	1.3 (0.8-2.1)	1.2 (0.6-2.3)	1.4 (1.0-2.0)	1.8 (1.3-2.6)	2.1 (1.6-2.8)	1.0 (0.5-2.2)	4.3 (1.6-11.8)	1.7 (1.4-2.0)
No	2.0 (1.5-2.6)	1.1 (0.6-1.9)	1.3 (0.6-2.6)	1.4 (1.0-2.1)	1.6 (1.0-2.4)	2.1 (1.6-2.8)	1.1 (0.5-2.4)	1.6 (0.8-3.0)	1.6 (1.4-2.0)
Hospitalization									
Yes	1.3 (0.8-2.0)	0.7 (0.3-1.4)	0.5 (0.2-1.7)	1.0 (0.7-1.7)	0.9 (0.5-1.7)	1.4 (1.0-2.0)	0.3 (0.1-1.3)	1.2 (0.6-2.3)	1.0 (0.8-1.3)
No	2.4 (1.7-3.4)	1.7 (0.9-3.1)	1.7 (0.7-3.9)	1.1 (0.6-2.2)	2.3 (1.4-3.6)	1.7 (1.0-2.7)	1.9 (0.8-4.8)	1.7 (0.6-4.7)	1.9 (1.5-2.5)

Abbreviations: ALK, anaplastic lymphoma kinase; BRAF, B-Raf proto-oncogene, serine/threonine kinase; CDK4/6, cyclin dependent kinase 4 or 6; eGFR, estimated glomerular filtration rate calculated using CKD-EPI 2021 creatinine equation; EGFR, epidermal growth factor receptor; *ERBB2*, human epidermal growth factor receptor 2; NA, not applicable; PARP, poly(adenosine diphosphate-ribose) polymerase; VEGFR, vascular endothelial growth factor receptor; sCr, serum creatinine.

^a^ Model adjusted for: hypertension, diabetes, ischemic heart disease, congestive heart failure, COPD, cirrhosis, age, sex, race, body mass index and eGFR before drug start.

^b^ Change in sCr indicates mean sCr value between 1 and 60 days of drug start minus the sCr value before drug start.

^c^ NA due to insufficient number of patients in cohort.

## Discussion

In this cohort study of 5015 patients who were treated with CDK4/6 inhibitors, PARP inhibitors, or select TKIs between 2008 and 2024, incidence and rates of progressive kidney dysfunction were higher among treated patients relative to a matched cancer-free cohort. Over a 2-year time frame, we observed that patients treated with specific TKIs (VEGFR inhibitors and EGFR inhibitors) and CDK4/6 inhibitors experienced higher incidence of progressive kidney dysfunction compared with their respective matched cohort. Relative rates of progressive kidney dysfunction matched these patterns, with higher relative rates additionally observed among patients treated with BRAF inhibitors and among subgroups without exposure to PPIs and potentially nephrotoxic chemotherapy or immunotherapy. Our analyses were anchored to a baseline sCr obtained in the early period after drug start—to account for the putative pseudoacute kidney injury described in the literature^2,4,37^—and thus our findings suggest a substantive risk for true kidney dysfunction among patients treated with specific molecularly targeted oral medications.

Meeting criterion for progressive kidney dysfunction as defined in our study implies an adverse kidney outlook. In an analysis of over 1.3 million persons with baseline eGFR>60 mL/min/1.73m^2^, as was the case for our study, a decline in eGFR of 30% or more over 2 years was associated with an approximately 7-fold higher relative rate of developing ESKD (HR, 6.7; 95% CI, 3.9-11.5).^33^ Between 3.4% and 5.5% of patients with a 30% or more decline in eGFR reached ESKD within 10 years, relative to less than 1% of patients without any change in eGFR.^33^ Although risk of ESKD may be less relevant for patients with metastatic cancer given shorter survival and limited treatment options, patients are living longer and therapies are moving to earlier stage cancers (eg, adjuvant osimertinib for stage IB-IIIA lung cancer). Therefore, knowledge on which medications are associated with kidney dysfunction is helpful because (1) newer medications may offer alternatives, (2) worsening kidney function could preclude other therapies, (3) stopping the medication could be considered (eg, adjuvant setting), and (4) interventions to prevent further decline could be used (eg, ACE inhibitors). At the same time, alternatives to these medications may carry a higher risk for severe complications and subsequent nephrotoxicity. Our goal with these analyses was to inform decision-making. Equipped with data quantifying the absolute and relative rate of progressive kidney dysfunction, clinicians can better counsel patients about the commonly observed and often distressing changes in sCr among patients treated with oral molecularly targeted therapies.

In our analysis, we selected drugs associated with an early change in sCr and/or reports of decreased creatinine secretion via OCT, OAT or MATE transporters since we hypothesized that an early change in sCr at drug start may help clinicians stratify risk for progressive kidney decline. Larger changes in sCr in response to secretion blockade could indicate diminished kidney function reserve.^38,39,40^ We found that for a majority of drug groups (with the possible exception of breakpoint cluster region-Abelson leukemia virus 1 inhibitors), the increase in risk was independent of this early change in sCr, and thus alternative methods are needed to risk stratify. Another serum measure of kidney function, cystatin C, is not affected by decreased creatinine secretion. Cystatin C values were rarely obtained in our cohort. With society guidelines recommending regular use of eGFR creatinine-cystatin C in patients with cancer,^41,42^ more patients may have this potentially informative measure of kidney function available in future studies.

The risk for progressive kidney dysfunction for patients treated with CDK4/6 inhibitors, VEGFR inhibitors, EGFR inhibitors, and BRAF inhibitors persisted even among patients not exposed to potentially nephrotoxic chemotherapy or immunotherapy and PPIs. Accumulating data identify PPIs as a major risk factor for CKD,^43,44^ and long-term use in patients with cancer should be carefully examined. The mechanism of kidney injury is well described only for 1 of the classes with an observed higher incidence or relative rate: VEGFR inhibitors. VEGF is essential for maintaining the integrity of the capillary and arteriole network, especially at the delicate capillary-podocyte interface.^45,46^ Monoclonal antibodies that inhibit VEGF and VEGFR inhibitors are associated with thrombotic microangiopathy, proteinuria, and CKD.^47,48^ Putative mechanisms for EGFR inhibitors, CDK4/6 inhibitors, and BRAF inhibitors nephrotoxicity are less clear. Several case reports for antibodies against EGFR report biopsy-proven glomerular disease.^15,16,17,49,50,51,52^ The effect of CDK4/6 inhibitors and BRAF inhibitors may be related to tubular injury based on limited biopsy reports.^14^ CDK4/6 inhibitors target regulators of cell cycle leading to cell cycle arrest. Similarly, BRAF inhibitors target a protein which regulates cell growth, division, differentiation, migration, and apoptosis,^53,54^ therefore, impaired cellular repair within the kidney could lead to progressive kidney dysfunction. Consistent with our results, a 12-month retrospective study^5^ of 474 women with breast cancer treated with CDK4/6 inhibitors reported a decline in eGFR among patients treated for the entire 12-month duration, although no patients reached the composite outcome of ESKD, eGFR<10, or sustained 40% decline in eGFR.

We did not observe higher incidence or relative rate of progressive kidney dysfunction in patients treated with PARP inhibitors compared with their matched cohort despite a reasonable sample size (570 patients) and 33% of patients experiencing a 20% or greater change in sCr at the time of drug start. These results were consistent with a 12-month retrospective study^3^ of 269 patients treated with PARP inhibitors, which reported a transient decline in eGFR that recovered upon drug cessation.

### Limitations 

Limitations of this study include reliance on EHR data, which may be missing or incomplete. Variables such as socioeconomic status, lifestyle factors, intercurrent symptoms, and social determinants of health are not fully captured in EHR. We also did not account for cancer stage or progression, computed tomography contrast, or antibiotics use. There remains potential for residual confounding as our matched cohort does not have cancer and this in itself may be protective for kidney disease, despite propensity score matching. Notably, our subgroup analyses demonstrate higher risks for progressive kidney dysfunction in the treated group even among those individuals without hospitalizations or exposure to additional nephrotoxic agents. Furthermore, the matched group had a higher rate of hypertension compared with the treated group (57.2% vs 42%), which also may have biased our results to the null if this represented a higher baseline risk for CKD. Body composition variability, more commonly in patients with cancer, may cause misclassification in eGFR decline, likely biasing analyses toward the null. Findings are based on aggregated data across drug groups; larger multicenter studies are needed to assess individual drug toxicity although notably we report the cohorts for longer-term kidney outcomes. Furthermore, we investigated selected drugs within each drug group that were associated with an early rise in sCr so our findings are not representative of the entire drug class. Finally, in this observational framework, causality cannot be established, and mechanistic studies are needed to identify pathways of drug nephrotoxicity.

Although EHR data have limitations, they also capture a clinical practice treated population and their associated outcomes and play an important role in pharmacovigilance with rare or late adverse effects that may not have been appreciated on trial. Kidney function related outcomes are amenable to EHR based assessment, as kidney function is monitored routinely and frequently in patients being treated for cancer. Additional strengths of our analyses include our verification of drug class classification, with the associated cancer diagnoses matching the indications of the class. Our primary outcome has been well-validated in terms of its association with adverse kidney health outcomes.

## Conclusion

In conclusion, we report incidence and relative rates of progressive kidney dysfunction in patients treated with oral molecularly targeted cancer medications that are associated with an early rise in sCr at the time of drug start. Although the known early rise in sCr may cloud the assessment, clinicians should carefully monitor kidney function trends in patients treated with CDK4/6 inhibitors, EGFR inhibitors, VEGFR inhibitors, and BRAF inhibitors.

## Notes

Supplement 1. eTable 1. Drugs Within the Treated Cohort Associated With Increased Serum Creatinine and/or INHIBITION of OCT, OAT, or MATE Transporters eTable 2. Categorization of Patients by Cancer Type Within Each Drug Class for Treated Cohort eTable 3. Baseline Urine Measurements by Drug Class in Treated Cohort eTable 4. Propensity-Matched Characteristics of Matched Cohort eTable 5. Medication Exposures for 2 Years Following Drug Start and Change in serum Creatinine Values Following Drug Start for Each Drug Class in Treated Cohort eTable 6. Incidence and Death Rate of Progressive Kidney Dysfunction Within Each Drug Class and in Matched Cohort eTable 7. Subdistribution Hazard Ratios of Progressive Kidney Dysfunction Overall and Within Each Drug Class Compared With Matched Cohort eTable 8. Association Between Potential Risk Factors for Progressive Kidney Dysfunction in Treated Cohort eFigure. Study Time Points When Serum Creatinine was Assessed eMethods 1. eMethods 2. eReferences.

Supplement 2. Data Sharing Statement
