# Errors in Key Points, Abstract, Results, Figures, and Tables

**DOI:** 10.1001/jamanetworkopen.2025.52290

**Published:** 2025-12-09

In the Original Investigation titled “Molecularly Targeted Cancer Medications and Kidney Health,”^1^ published November 6, 2025, there were errors in the Key Points, Abstract, Results, Figures, and Tables. Some instances of epidermal growth factor receptor (EGFR) were incorrectly identified as estimated glomerular filtration rate (eGFR). This article has been corrected.^1^
