# Supplementary material for: Molecularly Targeted Cancer Medications and Kidney Health
Source: JAMA Netw Open. 2025 Nov 6;8(11):e2541221. doi: 10.1001/jamanetworkopen.2025.41221 (PMC12593116; doi:10.1001/jamanetworkopen.2025.41221)
Supplement: Supplement 1. — eTable 1. Drugs Within the Treated Cohort Associated With Increased Serum Creatinine and/or INHIBITION of OCT, OAT, or MATE Transporters eTable 2. Categorization of Patients by Cancer Type Within Each Drug Class for Treated Cohort eTable 3. Baseline Urine Measurements by Drug Class in Treated Cohort eTable 4. Propensity-Matched Characteristics of Matched Cohort eTable 5. Medication Exposures for 2 Years Following Drug Start and Change in serum Creatinine Values Following Drug Start for Each Drug Class in Treated Cohort eTable 6. Incidence and Death Rate of Progressive Kidney Dysfunction Within Each Drug Class and in Matched Cohort eTable 7. Subdistribution Hazard Ratios of Progressive Kidney Dysfunction Overall and Within Each Drug Class Compared With Matched Cohort eTable 8. Association Between Potential Risk Factors for Progressive Kidney Dysfunction in Treated Cohort eFigure. Study Time Points When Serum Creatinine was Assessed eMethods 1. eMethods 2. eReferences. [file jamanetwopen-e2541221-s001.pdf]

Supplemental Online Content

Ziolkowski SL, Long J, Zhong Y, et al. Molecularly targeted cancer medications and kidney health. *JAMA Netw Open*. 2025;8(11):e2541221. doi:10.1001/jamanetworkopen.2025.41221

eTable 1. Drugs Within the Treated Cohort Associated With Increased Serum Creatinine and/or Inhibition of OCT, OAT, or MATE Transporters
eTable 2. Categorization of Patients by Cancer Type Within Each Drug Class for Treated Cohort
eTable 3. Baseline Urine Measurements by Drug Class in Treated Cohort
eTable 4. Propensity-Matched Characteristics of Matched Cohort
eTable 5. Medication Exposures for 2 Years Following Drug Start and Change in serum Creatinine Values Following Drug Start for Each Drug Class in Treated Cohort
eTable 6. Incidence and Death Rate of Progressive Kidney Dysfunction Within Each Drug Class and in Matched Cohort
eTable 7. Subdistribution Hazard Ratios of Progressive Kidney Dysfunction Overall and Within Each Drug Class Compared With Matched Cohort
eTable 8. Association Between Potential Risk Factors for Progressive Kidney Dysfunction in Treated Cohort
eFigure. Study Time Points When Serum Creatinine was Assessed
eMethods 1.
eMethods 2.
eReferences.

This supplemental material has been provided by the authors to give readers additional information about their work.

© 2025 Ziolkowski SL et al. *JAMA Network Open*.

eTable 1. Drugs Within the Treated Cohort Associated With Increased Serum Creatinine and/or INHIBITION of OCT, OAT, or MATE Transporters

Drug Class (inhibitors)	Drug name	Common target cancers
CDK 4/6	Abemaciclib,[1]<br>Ribociclib,[2]<br>Palbociclib[3]	Breast
PARP	Olaparib,[4-6]<br>Niraparib,[6,7]<br>Rucaparib[6]	Ovarian, breast, endometrial, prostate, pancreas
**Tyrosine Kinase inhibitors**		
ALK	Crizotinib,[8-10]<br>Alectinib,[11]<br>Ceritinib,[12]<br>Lorlatinib,[13]<br>Brigatinib[10]	Non-small cell lung
BCR-ABL	Imatinib [9,14]	Acute and chronic myeloid leukemia and gastrointestinal stromal tumor
EGFR	Geftinib,[13-18]<br>Osimertinib[13,19]	Non-small cell lung
VEGFR	Pazopanib,[9,20]<br>Sunitinib,[9,21]<br>Sorafenib[9,13]	RCC, sarcoma, gastrointestinal stromal tumor, hepatocellular, thyroid
ERBB2/HER2	Tucatinib,[22]<br>Lapatinib,[13]<br>Neratinib[23]	Breast
BRAF	Vemurafenib,[24]<br>Dabrafenib,[13]<br>Encorafenib[13]	Melanoma
MET	Capmatinib,[25,26]<br>Tepotinib[27]	Non-small cell lung

OCT= organic cation transporter, OAT= organic anion transporter, MATE= multi-drug and toxic compound extrusion proteins

© 2025 Ziolkowski SL et al. *JAMA Network Open*.

eTable 2. Categorization of Patients by Cancer Type Within Each Drug Class for Treated Cohort

Cancer site	Drug Class (inhibitors)								
	CDK 4/6	PARP	ALK	BCR-ABL	EGFR	VEGFR	ERBB2	BRAF	MET
n	1194	570	226	590	774	1131	218	282	30
Breast	1068 (89%)	135 (24%)					82 (82%)		
Genitourinary		76 (13.3%)				395 (35%)			
Gynecological		395 (69%)							
Lung			214 (95%)		765 (99%)				30 (100%)
Leukemia/ Lymphoma				317 (54%)					
Gastrointestinal or GIST				121 (21%)		542 (48%)		84 (30%)	
Melanoma								117 (42%)	
Head and Neck						33 (3%)		187 (66%)	
Other	109 (9%)	91 (16%)	8 (4%)	72 (12%)	1 (0.1%)	101 (9%)	36 (17%)	7 (3%)	
Unknown	6 (1%)	1 (0.2%)	3 (1%)	94 (16%)	2 (0.3%)	14 (1%)		3 (1%)	

Patients are categorized based on presence of diagnosis codes so can be classified into two cancer types.
GIST= gastrointestinal stromal tumor

© 2025 Ziolkowski SL et al. *JAMA Network Open*.

eTable 3. Baseline Urine Measurements by Drug Class in Treated Cohort

	Drug Class (inhibitors)								
	**CDK 4/6**	**PARP**	**ALK**	**BCR-ABL**	**EGFR**	**VEGFR**	**ERBB2**	**BRAF**	**MET**
n	**1194**	**570**	**226**	**590**	**774**	**1131**	**218**	**282**	**30**
24-hour urine count	5	3			1	3		1	
24-hour urine mean (mg)	163.3 (101.5)	622.3 (846.8)			110.0 (--)	646.0(851.4)		5829.0 (--)	
UPCR count	27	18	17	19	43	130	8	13	4
UPCR (g/g)	1.3 (4.6)	1.5 (2.3)	0.6 (0.5)	0.6 (0.6)	0.6 (1.7)	0.4 (0.6)	0.7 (0.6)	1.1 (1.8)	0.2 (0.1)
UACR count	44	14	5	20	26	36	6	8	2
UACR (g/g)	0.3 (1.1)	0.6 (1.4)	0.3 (0.5)	0.1(0.2)	0.1 (0.3)	0.4 (0.9)	0.3 (0.5)	0.6 (1.6)	0.02 (0.01)
Urinalysis count	360	334	91	257	247	697	95	147	14
Urinalysis with 2+ or higher count	21 (0.06%)	29 (0.09%)	1 (0.01%)	18 (0.07%)	18 (0.07%)	54 (0.08%)	8 (0.08%)	8 (0.05%)	3 (21.4%)

*data presented as n(%) or mean (standard deviation)
Counts represent how many patients had at least 1 measurement
UPCR= urine protein: creatinine ratio; UACR= urine albumin:creatinine ratio

© 2025 Ziolkowski SL et al. *JAMA Network Open*.

eTable 4. Propensity-Matched Characteristics of Matched Cohort

	Matched cohort (n=5,015)	Treated cohort (n=5,015)	Standardized mean difference Treated – Matched Cohort	P value
Matched variables				
Age, median (IQR)	64.0 (25.2)	62.1 (20.4)	0.037	0.063
Male, n (%)	1,700 (33.9%)	1751 (34.9%)	0.021	0.293
Race, n (%)				< 0.001
White	2,629 (52.4%)	2479 (49.4%)	0.061	
Black	251 (5.0%)	138 (2.8%)	<0.001	
Asian	1030 (20.5%)	1513 (30.2%)	0.223	
Other/unknown	1105 (22.0%)	886 (17.6%)	<0.001	
Diabetes	676 (13.5%)	850 (16.9%)	0.100	<0.001
Ischemic Heart Disease	331 (6.6%)	385 (7.7%)	0.047	0.021
Congestive heart failure	211 (4.2%)	244 (4.9%)	0.035	0.085
eGFR, ml/min per 1.73 m^2 (creatinine)	88.1 (25.2)	87.18 (23.29)	0.039	0.051
Additional variables				
eGFR Creatinine categories, n (%)				
>= 90	2,580 (51.5%)	2637 (52.6%)	0.005	
60-89	1,740 (34.7%)	1665 (33.2%)	0.011	
45-59	429 (8.6%)	448 (8.9%)	0.035	
30-45	180 (3.6%)	199 (4.0%)	0.015	
<30	86 (1.7%)	66 (1.3%)	0.036	
Hemoglobin	12.9 (1.9)*	12.3 (2.0)**	0.327	< 0.001
Albumin	3.9 (0.7)*	3.7 (0.7)**	0.366	< 0.001
Magnesium	2.6 (1.1)*	2.0 (0.3)**	0.695	< 0.001
Hypertension	2869 (57.2%)	2106 (42.0%)	0.300	< 0.001

*in matched cohort= hemoglobin available in 4700 patients, albumin available in 4827 patients, magnesium available in 386 patients
**in treated cohort: hemoglobin available in 4870 patients, albumin available in 4817 patients, magnesium available in 2576 patients

© 2025 Ziolkowski SL et al. *JAMA Network Open*.

eTable 5. Medication Exposures for 2 Years Following Drug Start and Change in serum Creatinine Values Following Drug Start for Each Drug Class in Treated Cohort

	Drug Class (inhibitors)								
	CDK 4/6	PARP	ALK	BCR-ABL	EGFR	VEGFR	ERBB2	BRAF	MET
n	1194	570	226	590	774	1131	218	282	30
Immunotherapy									
CTLA-4 inhibitors	1 (0.1%)	4 (0.7%)	0 (0.0%)	2 (0.3%)	0 (0.0%)	43 (3.8%)	0 (0.0%)	11 (3.9%)	0 (0.0%)
PD-1 inhibitors	40 (3.4%)	86 (15.1%)	12 (5.3%)	4 (0.7%)	32 (4.1%)	147 (13.0%)	2 (0.9%)	66 (23.4%)	5 (16.7%)
PD-L1 inhibitors	10 (0.8%)	7 (1.2%)	1 (0.4%)	0 (0.0%)	20 (2.6%)	2 (0.2%)	0 (0.0%)	1 (0.4%)	0 (0.0%)
Chemo- and cancer therapy:									
Cisplatin	1 (0.1%)	1 (0.2%)	2 (0.9%)	0 (0.0%)	4 (0.5%)	2 (0.2%)	0 (0.0%)	3 (1.1%)	0 (0.0%)
Pemetrexed	1 (0.1%)	4 (0.7%)	27 (11.9%)	0 (0.0%)	191 (24.7%)	2 (0.2%)	0 (0.0%)	5 (1.8%)	6 (20.0%)
Bevacizumab	10 (0.8%)	100 (17.5%)	13 (5.8%)	2 (0.3%)	80 (10.3%)	14 (1.2%)	8 (3.7%)	10 (3.5%)	2 (6.7%)
Gemcitabine	45 (3.8%)	60 (10.5%)	11 (4.9%)	2 (0.3%)	39 (5.0%)	61 (5.4%)	8 (3.7%)	2 (0.7%)	2 (6.7%)
Loop Diuretic Use	135 (11.3%)	88 (15.4%)	44 (19.5%)	120 (20.3%)	88 (11.4%)	277 (24.5%)	30 (13.8%)	35 (12.4%)	10 (33.3%)
NSAID use	257 (21.5%)	144 (25.3%)	41 (18.1%)	101 (17.1%)	128 (16.5%)	248 (21.9%)	44 (20.2%)	65 (23.0%)	5 (16.7%)
Proton Pump Inhibitor	349 (29.2%)	188 (33.0%)	84 (37.2%)	276 (46.8%)	267 (34.5%)	603 (53.3%)	91 (41.7%)	118 (41.8%)	13 (43.3%)
**Exposure to Drug Class (inhibitors)**									
Days, mean (SD)	535 (234)	511 (245)	539 (244)	570 (233)	514 (245)	490 (249)	515 (244)	417 (269)	441 (221)
Days, median (IQR)	672 (367, 731)	615 (299, 731)	730.5 (374, 731)	730.5 (407, 731)	617 (321, 731)	537 (298, 731)	616.5 (352, 731)	427 (163.5, 731)	441 (237, 618)
Patients on drug for ≥ 2 years	528 (44.2%)	227 (39.8%)	109 (48.2%)	318 (53.9%)	313 (40.4%)	392 (34.7%)	85 (39.0%)	76 (27.0%)	5 (16.7%)
**Change in serum creatinine at drug start**									
≥ 0.3 mg/dl change	100 (8.4%)	28 (4.9%)	16 (7.1%)	33 (5.6%)	29 (3.8%)	68 (6.0%)	8 (3.7%)	15 (5.3%)	6 (20%)
≥20% change	360 (30.2%)	151 (26.5%)	54 (23.9%)	92 (15.6%)	148 (19.1%)	150 (13.3%)	43 (19.7%)	49 (17.4%)	12 (40%)
Change, mean (SD)	0.08 (0.26)	0.07 (0.18)	0.04 (0.31)	0.02 (0.28)	0.03 (0.27)	0.01 (0.27)	0.06 (0.31)	0.01 (0.26)	0.09 (0.22)
Change, median (IQR)	0.06 (0.17)	0.06 (0.15)	0.05 (0.16)	0.00 (0.18)	0.04 (0.14)	0.00 (0.19)	0.02 (0.14)	0.00 (0.17)	0.02 (0.31)

sCr= serum creatinine

Change in sCr = mean sCr value between 1 and 60 days of drug start minus the sCr value before drug start.

© 2025 Ziolkowski SL et al. *JAMA Network Open*.

eTable 6. Incidence and Death Rate of Progressive Kidney Dysfunction Within Each Drug Class and in Matched Cohort

Inhibitor	Number of Events	Years at Risk	Event Rate (per 1000 person-years)	Death rate (per 1000 person-years)	Median Follow up, Days (IQR)
Overall Treated	**296**	**6748**	**44**	**186**	**561 (257, 730)**
CDK 4/6	72	1690	42	113	608 (321, 730)
PARP	23	777	29	203	576 (269, 730)
ALK	10	323	32	188	663 (334, 730)
BCR-ABL	35	859	41	86	731 (309, 730)
EGFR	42	1045	40	209	581 (255, 730)
VEGFR	92	1408	65	261	488 (202, 730)
ERBB2	6	296	21	234	577 (236, 730)
BRAF	13	314	40	338	401 (161, 730)
MET	4	34	120	333	421 (201, 630)
Matched	325	8509	38	15	731 (566, 730)

***Death ascertained from electronic health records**

© 2025 Ziolkowski SL et al. *JAMA Network Open*.

eTable 7. Subdistribution Hazard Ratios of Progressive Kidney Dysfunction Overall and Within Each Drug Class Compared With Matched Cohort

Inhibitor	Hazard Ratio	95% Confidence Interval	p-value
ALK	1.3	0.7 - 2.4	0.89
BCR-ABL	1.3	0.9 - 1.9	0.18
BRAF	1.2	0.7 - 2.2	0.21
CDK 4/6	1.7	1.3 - 2.3	<0.0001
EGFR	1.4	1.0 - 2.1	0.02
ERBB2	0.7	0.3 - 1.7	0.74
PARP	1.2	0.8 - 1.8	0.62
VEGF	1.7	1.3 - 2.2	<0.0001
Overall	1.4	1.2 - 1.6	<0.0001

© 2025 Ziolkowski SL et al. *JAMA Network Open*.

eTable 8. Association Between Potential Risk Factors for Progressive Kidney Dysfunction in Treated Cohort

Characteristic	Drug Class (inhibitors)								
	**CDK 4/6**	**PARP**	**ALK**	**BCR-ABL**	**EGFR**	**VEGFR**	**ERBB2**	**BRAF**	**Overall**
Immunotherapy	**3.2 (1.2 - 8.8)**	2.2 (0.8 - 6.3)	--	--	0.9 (0.2 - 4.2)	1.1 (0.6 - 2.3)	--	1.7 (0.5 - 6.4)	**1.6 (1.0 - 2.4)**
Cisplatin and/or pemetrexed	--	--	--	--	**3.6 (1.6 - 7.8)**	--	--	--	1.6 (0.9 - 2.8)
Gemcitabine and/or bevacizumab	1.2 (0.3 - 4.8)	2.0 (0.8 - 5.4)	4.2 (0.4, 45.3)	--	0.8 (0.2 - 2.5)	**2.9 (1.3 - 6.4)**	--	3.6 (0.4 - 35.8)	**1.8 (1.1 - 2.8)**
Proton Pump Inhibitors	1.3 (0.7 - 2.3)	2.4 (1.0 - 6.1)	3.3 (0.9, 12.2)	**4.5 (2.2, 9.5)**	**3.4 (1.8 - 6.5)**	**4.8 (2.9 - 8.0)**	0.5 (0.1 - 4.4)	3.0 (0.8 - 10.9)	**2.9 (2.3 - 3.7)**
Diabetes	1.2 (0.6 - 2.2)	0.6 (0.1 - 2.9)	--	0.9 (0.4, 2.1)	1.1 (0.5 - 2.4)	0.9 (0.6 - 1.6)	1.2 (0.1 - 11.5)	0.3 (0.0 - 3.0)	1.0 (0.8 - 1.4)
Ischemic heart disease	2.1 (1.0 - 4.7)	0.5 (0.1 - 3.8)	--	1.6 (0.5, 5.1)	1.6 (0.6 - 4.3)	1.0 (0.4 - 2.5)	2.3 (0.0 -118.6)	1.5 (0.2 - 14.6)	1.2 (0.8 - 1.8)
Congestive heart failure	0.9 (0.3 - 3.1)	2.2 (0.2 -20.2)	4.5 (0.4, 52.9)	1.6 (0.5, 5.2)	0.8 (0.2 - 3.9)	1.6 (0.7 - 4.0)	3.7 (0.2 - 84.6)	--	1.3 (0.0 - 2.1)
Chronic Obstructive Pulmonary Disease	--	--	1.3 (0.1, 12.1)	1.1 (0.3, 3.9)	0.6 (0.2 - 2.2)	0.5 (0.2 - 1.6)	--	3.2 (0.4 - 28.0)	0.6 (0.3- 1.2)
Cirrhosis	1.0 (0.1 - 8.0)	--	--	0.7 (0.1, 5.4)	NA	**1.8 (1.0 - 3.2)**	--	--	**1.6 (1.0 - 2.6)**
Genitourinary cancer	0.6 (0.1 - 2.5)	1.2 (0.4 - 3.9)	0.7 (0.1, 6.7)	--	0.6 (0.1 - 2.6)	0.8 (0.5 - 1.3)	--	3.1 (0.6 - 17.8)	0.8 (0.6 - 1.2)
Female Sex	1.2 (0.4 - 3.3)	0.7 (0.2 - 3.7)	0.5 (0.1, 2.6)	1.8 (0.9, 3.8)	0.8 (0.4 - 1.5)	08 (0.5 - 1.2)	--	0.46 (0.1 - 1.7)	0.98 (0.8 - 1.3)
Race (ref: White) Black	1.9 (0.6 - 6.3)	1.5 (0.2 -12.1)	--	1.8 (0.5, 6.3)	--	1.4 (0.5 - 3.6)	--	--	1.3 (0.7 - 2.3)
Asian	1.3 (0.7 - 2.5)	0.7 (0.2 - 2.1)	1.2 (0.3, 4.8)	0.4 (0.1, 1.2)	1.0 (0.5 - 2.0)	0.7 (0.4 - 1.3)	--	5.3 (1.0 - 29.4)	1.0 (0.7 - 1.3)
Other	1.0 (0.5 - 2.0)	0.5 (0.1 - 2.4)	--	1.1 (0.5, 2.6)	0.9 (0.3 - 2.7)	1.4 (0.8 - 2.4)	--	4.9 (0.9 - 27.4)	1.3 (0.9 - 1.7)
Body mass index (kg/m^2)	1.0 (1.0 - 1.1)	1.0 (1.0 - 1.0)	1.0 (0.9, 1.2)	1.0 (1.0, 1.1)	1.0 (1.0 - 1.0)	1.0 (1.0 - 1.0)	1.1 (0.9 - 1.3)	1.0 (0.9 - 1.1)	1.0 (1.0 - 1.0)
eGFR prior to drug start (ml/min/1.73m^2)	1.0 (1.0 - 1.0)	1.0 (1.0 - 1.0)	1.0 (1.0 - 1.0)	1.0 (1.0 - 1.0)	1.0 (1.0 - 1.0)	1.0 (1.0 - 1.0)	1.0 (1.0 -1.1)	1.0 (1.0 - 1.0)	1.0 (1.0 - 1.0)

© 2025 Ziolkowski SL et al. *JAMA Network Open*.

Change in sCr per 1 mg/dL	**0.5 (0.3 - 0.9)**	**0.1 (0.0 - 0.9)**	0.1 (0.0 - 9.3)	**3.7 (1.4 - 9.7)**	6.0 (0.7 - 49.7)	1.4 (0.6 - 3.2)	2.2 (0.0 - 445.5)	**10.6 (2.7 - 41.9)**	1.3 (0.8 - 2.1)
≥20% change in sCr*	0.8 (0.5 - 1.5)	0.3 (0.1 - 1.2)	0.2 (0.0 - 2.0)	**2.6 (1.1 - 5.8)**	0.8 (0.3 - 2.1)	1.2 (0.6 - 2.1)	--	2.9 (0.8 - 11.2)	1.0 (0.7 - 1.4)

sCr= serum creatinine
(--) signifies inadequate number of patients for evaluation
Change in sCr = mean sCr value between 1 and 60 days of drug start minus the sCr value before drug start.
Medication exposures were modeled as time-varying covariates in the model.
*tested in a separate model

© 2025 Ziolkowski SL et al. *JAMA Network Open*.

eFigure. Study Time Points When Serum Creatinine was Assessed

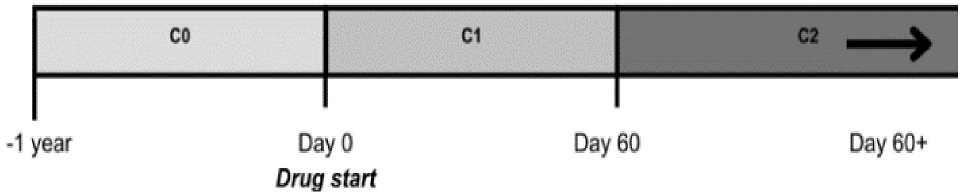

C0= creatinine prior to drug start (Table 1 values)
C1= creatinine day 1 to 60 after drug start date, mean eGFR calculated from these values was used as baseline for our analyses
C2= follow up serum creatinine values

© 2025 Ziolkowski SL et al. *JAMA Network Open*.

eMethods 1.

To determine the cancer type for each patient, we examined relevant condition concept sets from one year prior to two years after the drug start date. This approach ensured that only active cancers were included, while excluding historical or future diagnoses. We evaluated whether a patient had one of the most common cancers typically treated by each drug. Patients could be classified into two cancer types if relevant concept IDs were present for both cancers. All concept IDs are stored at https://github.com/ziolkows36/CKDOMOP.

eMethods 2.

To describe baseline urine measurements, we extracted all random and 24-hour urine albumin, urine protein and urine creatinine values obtained in the baseline period. All urine protein and albumin measurements were converted to milligrams. For urine protein to creatinine and urine to albumin creatinine ratios, measurements on the same day were paired to calculate ratios. All concept IDs are stored at https://github.com/ziolkows36/CKDOMOP.

© 2025 Ziolkowski SL et al. *JAMA Network Open*.

eReferences.

1. Chappell JC, Turner PK, Pak YA, et al. Abemaciclib Inhibits Renal Tubular Secretion Without Changing Glomerular Filtration Rate. *Clin Pharmacol Ther*. May 2019;105(5):1187-1195. doi:10.1002/cpt.1296

2. Sy-Go J, Yarandi N, Schwartz G, Herrmann S. Ribociclib-Induced Pseudo-Acute Kidney Injury. Journal of Onco-Nephrology2022. p. 64-69.

3. Gupta S, Caza T, Herrmann SM, Sakhiya VC, Jhaveri KD. Clinicopathologic Features of Acute Kidney Injury Associated With CDK4/6 Inhibitors. *Kidney Int Rep*. Mar 2022;7(3):618-623. doi:10.1016/j.ekir.2021.11.033

4. Bruin MAC, Korse CM, van Wijnen B, et al. A real or apparent decrease in glomerular filtration rate in patients using olaparib? *Eur J Clin Pharmacol*. Feb 2021;77(2):179-188. doi:10.1007/s00228-020-03070-0

5. McCormick A, Swaisland H. In vitro assessment of the roles of drug transporters in the disposition and drug-drug interaction potential of olaparib. *Xenobiotica*. Oct 2017;47(10):903-915. doi:10.1080/00498254.2016.1241449

6. Zibetti Dal Molin G, Westin SN, Msaouel P, Gomes LM, Dickens A, Coleman RL. Discrepancy in calculated and measured glomerular filtration rates in patients treated with PARP inhibitors. *Int J Gynecol Cancer*. Jan 2020;30(1):89-93. doi:10.1136/ijgc-2019-000714

7. Lazareth H, Delanoy N, Cohen R, et al. Nephrotoxicity Associated With Niraparib. *Am J Kidney Dis*. Dec 2020;76(6):898-900. doi:10.1053/j.ajkd.2020.05.021

8. Arakawa H, Omote S, Tamai I. Inhibitory Effect of Crizotinib on Creatinine Uptake by Renal Secretory Transporter OCT2. *J Pharm Sci*. 09 2017;106(9):2899-2903. doi:10.1016/j.xphs.2017.03.013

9. Omote S, Matsuoka N, Arakawa H, Nakanishi T, Tamai I. Effect of tyrosine kinase inhibitors on renal handling of creatinine by MATE1. *Sci Rep*. 06 18 2018;8(1):9237. doi:10.1038/s41598-018-27672-y

10. Tsang YP, López Quiñones AJ, Vieira LS, Wang J. Interaction of ALK Inhibitors with Polyspecific Organic Cation Transporters and the Impact of Substrate-Dependent Inhibition on the Prediction of Drug-Drug Interactions. *Pharmaceutics*. Sep 13 2023;15(9)doi:10.3390/pharmaceutics15092312

11. Pignataro D, Paratore C, Di Maio M, Tucci M, Novello S. Renal Function Change During Alectinib in ALK Rearranged Non-Small Cell Lung Cancer: A Retrospective Multicentre Analysis. Journal of Thoracic Oncology2021. p. S1090.

12. Soria JC, Tan DSW, Chiari R, et al. First-line ceritinib versus platinum-based chemotherapy in advanced ALK-rearranged non-small-cell lung cancer (ASCEND-4): a randomised, open-label, phase 3 study. *Lancet*. 03 04 2017;389(10072):917-929. doi:10.1016/S0140-6736(17)30123-X

13. Uddin ME, Talebi Z, Chen S, et al. In Vitro and In Vivo Inhibition of MATE1 by Tyrosine Kinase Inhibitors. *Pharmaceutics*. Nov 25 2021;13(12)doi:10.3390/pharmaceutics13122004

© 2025 Ziolkowski SL et al. *JAMA Network Open*.

14. Minematsu T, Giacomini KM. Interactions of Tyrosine Kinase Inhibitors with Organic Cation Transporters and Multidrug and Toxic Compound Extrusion Proteins. *Molecular Cancer Therapeutics*. 2011;10(3):531-539. doi:10.1158/1535-7163.Mct-10-0731
15. AstraZeneca Canada. Product monograph. Iressa gefitinib tablets. Available at: https://www.astrazeneca.ca/content/dam/az-ca/downloads/productinformation/iressa-product-monograph-en.pdf . Accessed August 13, 2018.
16. Latcha S, Jaimes EA, Gutgarts V, Seshan S. Case of Proteinuria, Worsening Hypertension, and Glomerular Endotheliosis With Erlotinib and Gefitinib. *Kidney Int Rep*. Nov 2018;3(6):1477-1481. doi:10.1016/j.ekir.2018.07.005
17. Kumasaka R, Nakamura N, Shirato K, et al. Side effects of therapy: case 1. Nephrotic syndrome associated with gefitinib therapy. *J Clin Oncol*. Jun 15 2004;22(12):2504-5. doi:10.1200/jco.2004.09.064
18. Maruyama K, Chinda J, Kuroshima T, et al. Minimal change nephrotic syndrome associated with gefitinib and a successful switch to erlotinib. *Intern Med*. 2015;54(7):823-6. doi:10.2169/internalmedicine.54.3661
19. Eide IJZ, Helland Å, Ekman S, Cicenas S. Abstract: Rapid drop in blood platelet count and increase in creatinine in non-small cell lung cancer (NSCLC) patients treated with osimertinib. Journal of Clinical OncologyMay 2018.
20. Sauzay C, White-Koning M, Hennebelle I, et al. Inhibition of OCT2, MATE1 and MATE2-K as a possible mechanism of drug interaction between pazopanib and cisplatin. *Pharmacol Res*. 08 2016;110:89-95. doi:10.1016/j.phrs.2016.05.012
21. Guo D, Yang H, Li Q, et al. Selective Inhibition on Organic Cation Transporters by Carvedilol Protects Mice from Cisplatin-Induced Nephrotoxicity. *Pharm Res*. Sep 6 2018;35(11):204. doi:10.1007/s11095-018-2486-2
22. Topletz-Erickson AR, Lee AJ, Mayor JG, et al. Tucatinib Inhibits Renal Transporters OCT2 and MATE Without Impacting Renal Function in Healthy Subjects. *J Clin Pharmacol*. 04 2021;61(4):461-471. doi:10.1002/jcph.1750
23. Alim K, Moreau A, Bruyère A, et al. Inhibition of organic cation transporter 3 activity by tyrosine kinase inhibitors. *Fundamental & Clinical Pharmacology*. 2021;35(5):919-929. doi:https://doi.org/10.1111/fcp.12657
24. Hurabielle C, Pillebout E, Stehlé T, et al. Mechanisms Underpinning Increased Plasma Creatinine Levels in Patients Receiving Vemurafenib for Advanced Melanoma. *PLoS One*. 2016;11(3):e0149873. doi:10.1371/journal.pone.0149873
25. Mohan A, Herrmann SM. Capmatinib-Induced Pseudo-Acute Kidney Injury: A Case Report. *Am J Kidney Dis*. 01 2022;79(1):120-124. doi:10.1053/j.ajkd.2021.04.009
26. Wolf J, Seto T, Han JY, et al. Capmatinib in. *N Engl J Med*. 09 03 2020;383(10):944-957. doi:10.1056/NEJMoa2002787
27. Paik PK, Felip E, Veillon R, et al. Tepotinib in Non-Small-Cell Lung Cancer with. *N Engl J Med*. 09 03 2020;383(10):931-943. doi:10.1056/NEJMoa2004407

© 2025 Ziolkowski SL et al. *JAMA Network Open*.
