# Supplementary material for: Molecularly Targeted Cancer Medications and Kidney Health
Source: JAMA Netw Open. 2025 Nov 6;8(11):e2541221. doi: 10.1001/jamanetworkopen.2025.41221 (PMC12593116; doi:10.1001/jamanetworkopen.2025.41221)
Supplement: Supplement 2. — Data Sharing Statement [file jamanetwopen-e2541221-s002.pdf]

Data Sharing Statement

Ziolkowski. Molecularly Targeted Cancer Medications and Kidney Health. *JAMA Netw Open*. Published November 06, 2025. doi:10.1001/jamanetworkopen.2025.41221

Data
Data available: Yes
Data types: Deidentified participant data
How to access data: The data will be made available after approval of a proposal, and with a signed data access agreement. Requests can be sent to : sziolkow@stanford.edu
When available: With publication

Supporting Documents
Document types: Statistical/analytic code
How to access documents: We created concept sets for laboratory measurements, demographics, medications, co-morbidities, and cancer types (available at https://github.com/ziolkows36/CKDOMOP).
When available: With publication

Additional Information
Who can access the data: This data is currently publicly available on github as stated.
Types of analyses: NA
Mechanisms of data availability: NA
